# Drug Combinations as a First Line of Defense against Coronaviruses and Other Emerging Viruses

**DOI:** 10.1128/mbio.03347-21

**Published:** 2021-12-21

**Authors:** Judith M. White, Joshua T. Schiffer, Rachel A. Bender Ignacio, Shuang Xu, Denis Kainov, Aleksandr Ianevski, Tero Aittokallio, Matthew Frieman, Gene G. Olinger, Stephen J. Polyak

**Affiliations:** a University of Virginia, Department of Cell Biology, Charlottesville, Virginia, USA; b University of Virginia, Department of Microbiology, Charlottesville, Virginia, USA; c University of Washington, Division of Allergy and Infectious Diseases, Seattle, Washington, USA; d Fred Hutchinson Cancer Research Center, Vaccine and Infectious Diseases Division, Seattle, Washington, USA; e Department of Clinical and Molecular Medicine, Norwegian University of Science and Technology, Trondheim, Norway; f Institute of Technology, University of Tartu, Tartu, Estonia; g Institute for Molecular Medicine Finland, FIMM, University of Helsinki, Helsinki, Finland; h Oslo Centre for Biostatistics and Epidemiology (OCBE), University of Oslo, Oslo, Norway; i Institute for Cancer Research, Oslo University Hospital, Oslo, Norway; j Department of Microbiology and Immunology, University of Maryland School of Medicine, Baltimore, Maryland, USA; k MRIGlobal, Gaithersburg, Maryland, USA; l Virology Division, Department of Laboratory Medicine and Pathology, University of Washington, Seattle, Washington, USA; m Department of Global Health, University of Washington, Seattle, Washington, USA; n Department of Microbiology, University of Washington, Seattle, Washington, USA; Albert Einstein College of Medicine

**Keywords:** SARS-CoV-2, COVID-19, viral pandemic, pandemic preparedness, antiviral drugs, drug synergy, model-driven approach, prophylaxis, early treatment, category A-C pathogens, Ebola virus, countermeasures

## Abstract

The world was unprepared for coronavirus disease 2019 (COVID-19) and remains ill-equipped for future pandemics. While unprecedented strides have been made developing vaccines and treatments for COVID-19, there remains a need for highly effective and widely available regimens for ambulatory use for novel coronaviruses and other viral pathogens. We posit that a priority is to develop pan-family drug cocktails to enhance potency, limit toxicity, and avoid drug resistance. We urge cocktail development for all viruses with pandemic potential both in the short term (<1 to 2 years) and longer term with pairs of drugs in advanced clinical testing or repurposed agents approved for other indications. While significant efforts were launched against severe acute respiratory syndrome coronavirus 2 (SARS-CoV-2), *in vitro* and in the clinic, many studies employed solo drugs and had disappointing results. Here, we review drug combination studies against SARS-CoV-2 and other viruses and introduce a model-driven approach to assess drug pairs with the highest likelihood of clinical efficacy. Where component agents lack sufficient potency, we advocate for synergistic combinations to achieve therapeutic levels. We also discuss issues that stymied therapeutic progress against COVID-19, including testing of agents with low likelihood of efficacy late in clinical disease and lack of focus on developing virologic surrogate endpoints. There is a need to expedite efficient clinical trials testing drug combinations that could be taken at home by recently infected individuals and exposed contacts as early as possible during the next pandemic, whether caused by a coronavirus or another viral pathogen. The approach herein represents a proactive plan for global viral pandemic preparedness.

## INTRODUCTION

The nucleotide sequence for severe acute respiratory syndrome coronavirus 2 (SARS-CoV-2), the virus that sparked the global coronavirus disease 2019 (COVID-19) pandemic, was released in January 2020. The scientific community rallied with dedication, efficiency, and skill such that novel vaccines and therapeutic antibodies demonstrated safety and efficacy in clinical trials and were authorized for administration by December 2020. Nevertheless, and despite the very recent arrival of promising antiviral drugs, the scale of death and illness as well as economic and social havoc remains unprecedented. The lack of readily available, widely implemented therapies with utility before the onset of severe complications continues to contribute to high global mortality. For the present pandemic response, and for future pandemics, whether from a coronaviruses or another virus, the scientific community must be ready with an arsenal of easily self-administered drugs (i.e., oral or inhaled) that can be tested in rapid, efficient clinical trials immediately after the causative viral agent is identified. We propose a proactive drug development strategy for high consequence viral pathogens that focuses on combinatorial approaches.

## OVERVIEW: DEVELOPING ANTIVIRAL REGIMENS FOR THE NEXT GLOBAL VIRAL PANDEMICS

Since it is highly likely that pathogenic viruses will continue to spill over into humans, a major component of pandemic preparedness should be development of highly effective and widely available antiviral treatments for nonhospitalized patients. In our opinion, we must develop concurrent plans for two possible scenarios: plan A for the longer term whereby the next serious viral outbreak does not occur for ∼5 to 10 years and plan B for the dire possibility that an epidemic arises within ≤1 to 2 years, from a new CoV or from a high consequence virus currently lacking effective self-administered antiviral drugs. Both plans should focus on drug combinations, but potential input drugs would differ. Plan A would focus on the development of new chemical entities (NCEs), in particular directly acting antivirals (DAAs). Plan B could employ DAAs and host-targeting agents (HTAs), including repurposed drugs and drugs in advanced clinical testing.

**FIG 1:**
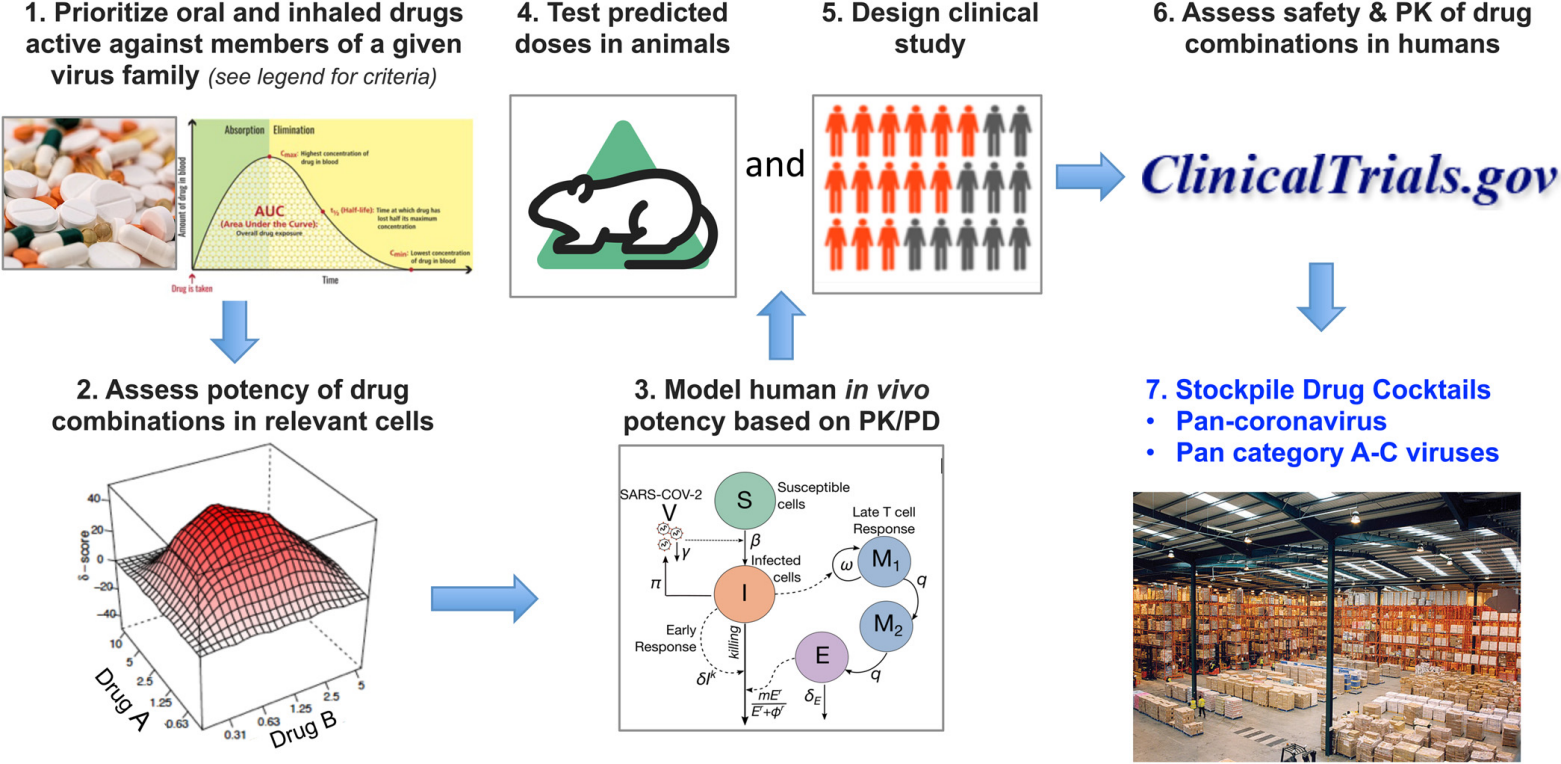
A model-driven approach to develop highly potent drug combinations for global viral pandemic preparedness. The same pipeline can be used to prepare for the long term (plan A [5 to 10 years to the next outbreak]) or short term (plan B [<1 to 2 years to the next outbreak]). (Step 1) Select drugs that can be delivered orally or, for respiratory viruses, via inhalation that (i) are approved or in advanced clinical trials for plan B, with drugs in development additionally included for plan A, (ii) are active in relevant human cells, (iii) are ideally DAAs, but HTAs can also be considered, (iv) have relatively high selectivity indices (SI) (CC_50_/EC_50_), and (v) have relatively high *C*_max_/EC_50_ or maximum target tissue concentration/EC_50_. (Step 2) Test pairs of priority drugs (drug A and drug B) for combination effects (e.g., multiplicative or synergistic) in relevant human cells using checkerboard assays. For respiratory viruses, this should include lung cell models such as Calu3 as well as a three-dimensional (3D) culture such as lung organoids or primary human airway epithelial cells at an air-liquid interface. Drug combinations should then be prioritized for advancement based on the following: (i) drug levels needed for virus inhibition; (ii) SI; (iii) effectiveness over the entire dose-response matrix, including whether the drugs act synergistically; (iv) differing targets; (v) resistance map profiles; and (vi) other PK parameters (e.g., drug-drug interactions, side effects, half-lives, protein binding). (Step 3) Model the potential for top combinations to be potent in humans based on known PK and PD. (Step 4) Test most promising pairs of oral (and/or inhaled) drugs in small animal models. (Step 5 [concurrently with step 4]) Design clinical study. (Step 6) Conduct phase 1 trial of the drug combination. The deliverables (Step 7) will be pan-virus family oral/inhaled drug cocktails that can be stockpiled and ready for use very early following identification of the family of a pandemic-causing virus. The predesigned clinical study can be immediately implemented in the face of an on-going pandemic. The name VORTEC (Viral Outbreak Readiness Through Effective Combinations) has been suggested for the approach. The image in Step 1 (right) is from https://clinicalinfo.hiv.gov/en/glossary/pharmacokinetics. The image in Step 3 is reprinted from reference 16 with permission (© The Authors, some rights reserved; exclusive licensee AAAS. Distributed under a CC BY-NC 4.0 license [http://creativecommons.org/licenses/by-nc/4.0/]). Other images are from https://commons.wikimedia.org/wiki/Main_Page.

For both scenarios, a successful drug development program would be based on five key tenets (Fig. 1). The first is to prioritize oral and inhaled drugs that could be taken at home as postexposure prophylaxis or early during illness to rapidly lower viral loads and subsequent harmful immune activation. The second is to search for drug combinations to reduce emergence of drug-resistant mutants and, through multiplicative or synergistic effects, bring needed drug doses into therapeutic range and mitigate side effects associated with high doses of single drugs. The third is to prioritize drugs that are approved or in advanced clinical testing to allow a more rapid regulatory review process. Plan A with the relative luxury of time would also include drugs currently in preclinical development. The fourth is to prioritize drugs whose effective concentrations in relevant human tissue models are considerably below their toxic concentrations and in the range of attainable levels throughout the dosing interval. The fifth is to employ mathematical modeling at critical junctures of advancing to animal testing and clinical trial design. The products would be combinations of drugs that could be deployed widely and early during an outbreak as prophylaxis and early treatment. In conjunction with other nonpharmaceutical interventions, such in-home use drug cocktails could limit the burden on health care systems and thwart person-to-person virus spread by lowering viral loads and the virus’ ability to adapt to the host.

## CURRENT STATE OF SARS-CoV-2 THERAPEUTICS

Early during the current pandemic, there were no therapeutics against SARS-CoV-2, and slight improvements in clinical outcomes were linked solely to advances in supportive care. Then, in May 2020, remdesivir (RDV), a SARS-CoV-2 polymerase inhibitor (1, 2), received an emergency use authorization (EUA) (3–8) for intravenous (IV) administration in hospitalized patients, with significant benefits noted in recent reports (3, 8). A very recent trial of IV RDV showed an 80% reduction in hospitalization (9), highlighting that treatment soon after infection is likely to be more efficacious than treatment later in the course of illness during hospitalization. Dexamethasone, an immunosuppressant without antiviral effects, also received EUA for clinical benefit in severe or critically ill hospitalized patients (10). Another set of drugs with an EUA for IV administration is RDV plus baricitinib (see below).

In November 2020, the first two monoclonal antibodies (MAbs), which target the SARS-CoV-2 spike glycoprotein, received an EUA after showing reductions in hospitalization and severe illness when dosed in clinical trials during early illness (11–15). MAbs are now available in limited settings for early use in high-risk patients, but their requirement for parenteral administration has precluded widespread implementation early during infection when therapy is most likely to be effective (16) Other limitations of MAbs include cost, manufacturing capacity, and emergence of antibody-resistant variants (17), which has already eliminated the utility of one promising MAb.

An exciting step forward is therefore the arrival of two oral SARS-CoV-2 drugs: the oral polymerase inhibitor molnupiravir (MPV, EUA approved in Europe and pending in the United States [18–21]), and the oral protease inhibitor Paxlovid (22) (PF-07321332/ritonavir; EUA pending). MPV elicited a 30% reduction in hospitalizations and death in infected high-risk people diagnosed and treated within 5 days of symptom onset (https://www.merck.com/news/merck-and-ridgeback-statement-on-positive-fda-advisory-committee-vote-for-investigational-oral-antiviral-molnupiravir-for-treatment-of-mild-to-moderate-covid-19-in-high-risk-adults/), while Paxlovid reduced hospitalizations and death by 89% when administered within 3 days of symptom onset and showed similar benefit if given within 5 days. Many other oral/inhaled anti-SARS-CoV-2 drugs are in the pipeline. Nevertheless, these pivotal strides are occurring almost 2 years into the pandemic and after 5 million people have died. During the next pandemic, be it of a novel CoV or another virus, a major priority must be to discover and widely distribute effective outpatient treatment regimens within weeks rather than years.

## SCREENS FOR DRUGS TO THWART SARS-CoV-2 INFECTIONS

In the early days of the pandemic, work on small molecule antiviral drugs suffered from lack of a centralized research structure and strategic plan designed to keep pace with a rapidly expanding pandemic (23, 24). Many screens were conducted to identify drugs with activity against SARS-CoV-2 (25–32), and at least 216 small molecules approved by the Food and Drug Administration (FDA) have been reported to block SARS-CoV-2 in cell cultures. Yet most were identified in a convenient and nonrepresentative cell line (Vero E6 kidney) and later shown to have limited potency in lung epithelial cells (28, 33), the relevant cell type for SARS-CoV-2 infection and pathogenesis (34–36): SARS-CoV-2 enters Vero E6 cells by fusion in endosomes, whereas it enters lung cells by fusion at the cell surface following activation of the spike glycoprotein by the host cell surface serine protease TMPRSS2 (34–41). Many of the Vero E6 drug hits affect endosomes and were therefore not expected to block entry of SARS-CoV-2 into lung epithelial cells (38, 42). In addition, many had not completed safety and efficacy trials or are available only for IV use, making rapid widespread implementation infeasible. Many hits were also insufficiently potent against SARS-CoV-2 in lung cells *in vitro* at nontoxic doses; either their selective indices (SI), the ratio of half maximal efficacy to half lethal concentrations (EC_50_/CC_50_) were too low, or they would not achieve sufficient peak concentrations (*C*_max_) following standard dosing, reflected in a low *C*_max_/EC_50_ ratio.

These limitations contributed to the failure of many solo agents tested in clinical trials, including the highly publicized agents hydroxychloroquine (5, 30, 31) and ivermectin (43). While oral drugs to lower the risk of COVID-19-associated hospitalization and death are imminently available (18, 22) (see below), we urge the development of drug combinations to prevent the emergence of SARS-CoV-2 drug-resistant mutants (44, 45) and to potentially increase potency and breadth of coverage, and to reduce side effects.

## HIGHLY EFFECTIVE DRUG COMBINATIONS AGAINST OTHER VIRUSES

We advocate for developing combinations of oral, intranasal, and inhaled drugs to prepare for emerging and reemerging viral pandemics (46–48) based on precedent with other viral infections. Treatments for chronic illnesses caused by human immunodeficiency virus (HIV) and hepatitis C virus (HCV) (49–51) are composed of two to four oral DAAs that target multiple steps in the viral life cycles, thereby inducing multiplicative or synergistic antiviral effects (50–53). The combinations reduce doses of the individual drugs needed (50), thus lowering toxic side effects. (Certain small molecules may not provide synergy but instead enhance levels of other active agents. For HIV, cobicistat and ritonavir are coformulated with integrase inhibitors and protease inhibitors, respectively, to limit metabolization of the primary agent allowing lower doses [54, 55] [also see NCT04960202].) In addition, by targeting separate steps with distinct escape mutations, successful combination regimens eliminate selection of drug-resistant viruses (56–59). While emergence of drug resistance is less certain for acute viral infections, which are usually eliminated by the acquired immune response (16), SARS-CoV-2 and influenza infections in immunocompromised hosts are notable for prolonged viral persistence at high viral loads with considerable ongoing viral mutagenesis (60–65). Thus, developing combination therapies for acute viral infections is justified, and efforts are under way for influenza virus (66), Ebola virus (47, 48, 67, 68), arenaviruses (69), and SARS-CoV-2. For Ebola virus, computational modeling (48) has suggested that combinations would provide superior *in vivo* activity to their single agent components.

## CURRENT PROGRESS ON DRUG COMBINATIONS AGAINST SARS-CoV-2

There have been at least 34 reports of small molecule drug combinations against SARS-CoV-2, comprising a total of 77 unique drug pairs (26, 70–100, 197–199). Many were identified in Vero E6 cells, while others were found in Calu3 lung epithelial cells. Sixty-two pairs include RDV (101), with the partner drug being arbidol, BAY-2402234, brequinar, budesonide, camostat, cepharanthine, cilostazol, clobetasol, clofazimine, cobicistat, conivaptan, dabrafenib, diltiazem, drosiprenone, emetine, ezetimibe, interferon alpha, imino sugars, IQ-1S, ivermectin, ivosidenib, lapatinib, lenvatinib, linoleic acid, mefloquine, meprednisone, MU-UNMC-2, nelfinavir, nifedipine, nimodipine, nitazoxanide, omeprazole, omipalisib, pioglitazone, quinapril, raloxifene, reserpine, rifaximin, sangivamycin, selexipag, stenoparib, sulforaphane, telmisartan, tetrandrine, tipifarnib, valdecoxib, and zafirlukast.

RDV has also been reported to synergize with six oral HCV drugs (elbasvir, grazoprevir, paritaprevir, simeprevir, vaniprevir, and velpatasvir) as well as the HCV oral combination drugs Epclusa (velpatasvir plus sofosbuvir) and Zepatier (elbasvir plus grazoprevir) (75, 79, 89). These drugs block the HCV nonstructural serine protease (HCV NS3/4A), the HCV replication-associated protein NS5A, or the HCV polymerase (NS5B). The HCV drugs likely inhibit related functions of SARS-CoV-2 proteases and replication machinery, albeit at significantly lower potency. Four HCV protease inhibitors that synergized with RDV in Vero E6 cells (simeprevir, vaniprevir, paritaprevir, and grazoprevir) block the SARS-CoV-2 papain-like protease (PL^pro^), a cysteine protease encoded by nsp3 of SARS-CoV-2 (75). Two HCV protease inhibitors (boceprevir and narlaprevir) that blocked the SARS-CoV-2 C-like protease (3CL^pro^ or M^pro^, a cysteine protease encoded by SARS-CoV-2 nsp5), were inhibitory on their own (EC_50_ for boceprevir, 20 to 40 μM; EC_50_ for narlaprevir, 8 to 37 μM, in Vero E6 cells) (75, 89, 102) but did not synergize with RDV (102). Experimental drugs, including brilacidin (88), a 3CL^pro^ inhibitor (81), a RAD51 inhibitor (87), the natural product angeloylgomisin O (95), and a vitamin E derivative (97) have also been shown to synergize with RDV.

Synergistic drug pairs that do not involve a polymerase inhibitor have also been identified in Vero E6 cells, including nelfinavir plus amodiaquine (71), arbidol plus nitazoxanide (73), nitazoxanide plus emetine (73), and nelfinavir plus cepharanthine (78). Additional non-polymerase-targeted pairs found synergistic in Calu3 cells were interferon alpha plus camostat (86), interferon alpha plus nafamostat (96), and apilimod plus camostat (103). Interferon alpha plus nafamostat reduced viral loads in hamsters to a greater extent than the individual component drugs (96).

Viral polymerases are clearly good targets for DAAs, and 43 drugs have been reported to synergize with the polymerase inhibitors MPV and/or RDV. Given the goal of developing a regimen for outpatient use, we highlight in Table 1 oral and inhaled drugs that synergistically impede SARS-CoV-2 in Calu3 epithelial cells in conjunction with MPV, the oral polymerase inhibitor (18–21): three orally available drugs, the pyrimidine biosynthesis inhibitors BAY-2402234 and brequinar (77) and the HIV (aspartic) protease inhibitor nelfinavir (80), and the inhaled drug interferon alpha. We also list in Table 1 drugs reported to synergize in Calu3 cells with RDV. Although not a certainty, these drugs may also synergize with MPV, as both drugs target the SARS-CoV-2 RNA-dependent RNA polymerase (2, 104), albeit with different biochemical mechanisms (19, 105). Indeed, similar synergistic activity of RDV and MPV drug pairs was reported in two studies (77, 86). Of the 14 drugs listed, 9 are approved by the FDA, and 2 are used in Japan. Nine of the approved drugs are formulated for oral use; interferon alpha and ciclesonide are inhaled therapeutics. Two other agents appeared to synergize with RDV in Calu3 cells but were tested only at two doses: the approved oral drug mefloquine (98) and the investigational drug brilacidin (88). Of the pairs in Table 1, only the combination of MPV plus brequinar has been tested in an animal model, where it reduced viral loads and lung pathology favorably compared to either drug alone (77). Brequinar has demonstrated synergy against HCV in combination with sofosbuvir, an orally available HCV polymerase inhibitor (80).

**TABLE 1:** Drugs reported to synergize with remdesivir or molnupiravir to inhibit SARS-CoV-2 infection of Calu3 lung cells^*a*^

Drug A	Drug B	Drug B: FDA status^*b*^	Drug B CoV CT^*b*^	Drug B oral	Drug B target^*c*^	Drug B: step blocked^*c*^	Reference(s)^*d*^
Remdesivir (approved for intravenous use for COVID-19)	Nelfinavir	HIV	Ph2^*e*^	Yes	M-Pro^*f*^	Cleavage	80, 94
	Velpatasvir	HCV	Ph2^*g*^	Yes	Pol complex^*f*^	Replication	79
	Elbasvir	HCV	No	Yes	Pol complex^*f*^	Replication	79
	Grazoprevir	HCV	No	Yes	PL-Pro^*f*^	Cleavage	79
	Dabrafenib	Cancer	No	Yes	B-Raf kinase	ND^*h*^	79
	Cilostazol	Leg pain	No	Yes	PDE III	ND	79
	Nimodipine	Aneurysm	No	Yes	Ca channels^*i*^	ND	79
	Interferon alpha	HBV, HCV	Ph3	No	ISGs	Replication^*j*^	86
	B02	Preclin.	No	NA	RAD51	ND	87
	Camostat	Preclin.^*k*^	Ph3	Yes	TMPRSS2	Fusion	94
	Cepharanthine	Preclin.^*k*^	No	Yes	Multiple	ND^*k*^	94
	Ciclesonide	Rhinitis	Ph3	No	nsp3/4^*l*^, GlucR	Replication^*l*^	94
	Brequinar	Ph2 (AML)	Ph2	Yes	DHODH^*m*^	Replication	77
	BAY-2402234	Ph1 (MM)^*n*^	No	Yes	DHODH	Replication	77

Molnupiravir (EUA in Europe/pending in U.S. for oral use for COVID-19)	Nelfinavir	HIV	Ph2^*e*^	Yes	M-Pro^*f*^	Cleavage	80
	Interferon alpha	HBV, HCV	Ph3	No	ISGs	Replication^*j*^	86
	Brequinar	Ph2 (AML)	Ph2	Yes	DHODH	Replication	77
	BAY-2402234	Ph1 (MM)^*n*^	No	Yes	DHODH	Replication	77

a Abbreviations: AML, acute myeloid leukemia; CT, clinical trial; DHODH, dihydroorotate dehydrogenase; FDA, Food and Drug Administration; GlucR, glucocorticoid receptor; HBV, hepatitis B virus; HCV, hepatitis C virus; HIV, human immunodeficiency virus; ISGs, interferon-stimulated genes; MM, multiple myeloma; NA, not available; ND, not determined; PDE, phosphodiesterase; Ph, phase; Pol, polymerase; preclin., preclinical.

b Unless specified, drugs are FDA approved for the indicated conditions. Drugs in phase 1 (Ph1) for COVID-19 (CoV), e.g., reference 97, are not listed.

c Known or inferred target. Cleavage denotes polyprotein cleavage.

d References are for reported synergies. Reference 79 reports 15 additional remdesivir synergies, but the cell type analyzed was not specified.

e The https://biorender.com/covid-vaccine-tracker website lists a phase 2 (Ph2) study, but this was not in https://clinicaltrials.gov/.

f See text and references 75, 78, and 79 for targets of nelfinavir, velpatasvir, elbasvir, and grazoprevir.

g A trial (IRCT20130812014333N145) of Epclusa (sofosbuvir/velpatasvir) deemed it safe but of no apparent benefit.

h Dabrafenib inhibits lymphocytic choriomeningitis virus replication (32).

i L-type Ca channels.

j Proposed in reference 86.

k Camostat and cepharanthine are used in Japan for pancreatitis and multiple ailments, respectively (196).

l Ciclesonide is an anti-inflammatory, but reference 144 supports additional action versus SARS-CoV-2 nsp3 and nsp4.

m DHODH is a host enzyme required for pyrimidine biosynthesis.

n The trial in patients with advanced myeloid malignancies (NCT03404726) was terminated due to lack of clinical benefit.

Two drugs are registered in clinical trials in combination with RDV: camostat (NCT04713176) and the Janus kinase inhibitor, baricitinib (NCT04401579 and NCT04693026). Although baricitinib has not been shown to synergize with RDV *in vitro*, it provided moderate clinical benefit when added to RDV therapy (106), supporting its EUA for hospitalized COVID-19 patients. On the basis of the results of *in vitro* studies, baricitinib is hypothesized to provide benefit by both antiviral and anticytokine effects (107), and its efficacy in clinical use may be independent of RDV coadministration (108). In another clinical trial (109), administration of two oral HIV protease inhibitors (lopinavir and ritonavir), an oral broad-spectrum viral replication inhibitor (ribavirin), and an injected anti-inflammatory (interferon beta-1b) reduced viral loads and improved symptoms in hospitalized patients compared to lopinavir/ritonavir alone. While these studies highlight that combinations of DAAs and immunomodulatory medicines might have a therapeutic role, their need for parenteral administration precludes easy use during early infection when DAAs have the highest potential to limit disease severity.

## DESIGNING EFFECTIVE DRUG COMBINATIONS

As for oral drug combinations for patients with HIV and HCV, an ideal cocktail would contain multiple agents targeting CoV proteins, i.e., multiple DAAs. A theoretical cocktail might target the spike glycoprotein, the polymerase, and either or both viral proteases. We contend that HTAs should also be considered (47, 48, 69), perhaps to bolster pairs of DAAs. The combination of a protease inhibitor (HTA, aprotinin, given IV) targeting the host enzyme TMPRSS2, plus oral favipiravir (DAA, polymerase inhibitor) has been tested in COVID-19 patients, but the study was too small to assess clinical efficacy (110). In murine models of infection, combinations of two anti-Spike MAbs plus RDV provided benefit over single agents in some measures of COVID-19 disease (111), supporting the concept of combined targeting of virus entry and viral polymerase activity.

A current priority is to develop an organized structure to assess pairs of agents comprehensively and strategically. As shown above, there are many possible agents to consider for this purpose (112, 113). Below we propose criteria for prioritizing combinations, noting that new drugs should be incorporated into this schema as they become available.

### Identify drug pairs with enhanced combinatorial potency.

Most drug combination studies involve a checkerboard assay in which various doses of drugs A and B are set in a matrix, and these defined dose mixtures (36 for a 6 × 6 matrix) are tested for inhibition of SARS-CoV-2 infection (step 2, Fig. 1). These assays assess whether drug interactions are antagonistic, neutral, additive, multiplicative, or synergistic, in increasing order of desirability. While synergy is always in theory preferable, its highest potential is in the context of single agents that have insufficient potency on their own, as may be the case for repurposed agents. Antagonistic pairs should generally be excluded from further consideration. For interstudy comparisons, it would be ideal if future studies were coordinated to employ the same virus strains, target cells, infection protocol, assay readout, reference drug pairs, and synergy scoring methodology. While many DAAs should function independently of cell type, testing in lung cell systems (e.g., Calu3 cells, human airway epithelial cells cultured at the air-liquid interface, or lung organoids [34, 114, 115]) will be especially important for drugs that target entry, which varies by cell type (28, 33–36). Promising combinations should also be tested against the most relevant emerging SARS-CoV-2 variants of concern (VOCs, e.g., omicron) and other CoVs.

SynergyFinder (https://synergyfinder.fimm.fi/) is a publicly available web tool (116) that can be used to assess whether a drug pair tested in a checkerboard assay is considered synergistic. The program can assess synergy according to Bliss independence, zero interaction potential (ZIP) or Loewe additivity mathematical models. Synergy occurs when observed potency exceeds that of the expected combination effect based on the selected synergy model. In the strictest sense, synergy denotes observed potency exceeding that predicted by a Bliss independence mathematical model, which assumes multiplicative effects of paired drugs (50). With SynergyFinder, synergy scores are calculated over the full dose matrix as well as its maximum synergistic area (MSA); scores of >10 are generally considered synergistic (69, 116, 117). MSA ZIP scores for the HCV drugs listed in Table 1 combined with either RDV or MPV, based on assays that monitored virus-induced cytopathic effect in Calu3 cells, ranged from 50 to 85, with reported MSA ZIP scores for other pairs ranging from 22 to 52 (not given for brilacidin or the pairs with pyrimidine biosynthesis inhibitors). Thus, there now exist effective methods and freely available tools to identify synergistic drug combinations.

### Consider the molecular target of the drug and how the drug impinges on the SARS-CoV-2 life cycle.

A useful cocktail would contain two (or three) DAAs that target distinct CoV proteins. These may encompass drugs targeting the spike protein to block receptor binding or fusion or the enzymatic activities of its polymerase or proteases. The justification for this conclusion is based on comprehensive combinatorial testing of all licensed HIV antivirals (50). Compounds that target the same viral protein tend to have only additive effects in which the second agent adds little to overall potency. Molecules that distinctly target HIV reverse transcriptase, integrase, or protease usually have either multiplicative or synergistic effects and bypass resistance (59), though mechanisms that predict synergy rather than simply multiplicative effects are less clear from these data sets. HTAs that limit viral replication (31, 118–120) may enhance synergy of a set of DAAs and should be considered in the testing matrices.

### Consider the human exposure potential of the drugs.

To leverage pairwise synergy for potential therapeutic use, the pharmacokinetic (PK) profiles of component drugs are critical. Of importance are peak and trough drug concentrations, which are determined by tissue clearance kinetics. Ideally, both drugs achieve levels that allow Bliss independence or synergy throughout the dosing interval. If there are only brief time windows of drug synergy, then synergy may have limited beneficial effects for infections such as SARS-CoV-2 that are defined by rapid replication and spread dynamics (16). For example, we demonstrated that acyclovir only partially lowers genital herpes simplex virus 2 (HSV-2) shedding rates because 6-h windows of subtherapeutic drug levels prior to redosing are sufficient for breakthrough viral replication (121).

It is important to note that synergy may provide limited added benefit in situations where a given concentration of a single agent already eliminates nearly 100% of new cell infections. This possibility highlights the need to factor in projected peak and trough concentrations of both relevant drugs relative to their EC_50_s to assess whether multiplicative or synergistic pharmacodynamics (PD) provides meaningful additional benefit.

For MPV, the *C*_max_ at the dose of 800 mg given twice daily is 14 μM, while its EC_50_ against SARS-CoV-2 in Calu3 cells is 0.08 μM (19). This gives a *C*_max_/EC_50_ ratio of 175, meaning that a serum concentration well above its *in vitro* EC_50_ against SARS-CoV-2 is expected. Indeed, recent trial results indicate that MPV can achieve sufficient levels to lower viral replication (18), though drug synergy may further enhance its *in vivo* potency. As a note of caution, we have demonstrated that *in vitro* EC_50_ values often overestimate antiviral potency *in vivo* such that in order to suppress virus in people, 5 to 10 times higher drug levels are required than predicted by cell culture assays (122). It is also critical to consider each drug’s binding to protein *in vitro* and *in vivo*, as this can greatly influence the amount of drug available (123, 124).

Of the 11 oral drugs listed in Table 1 for which sufficient data are available, only 3 have *C*_max_/EC_50_ ratios approaching or greater than 1, which could be considered a minimal criterion for consideration due to the pharmacologic factors described above. They are camostat, nelfinavir, and brequinar, with *C*_max_/EC_50_ values of ∼1, 7, and 33, respectively. Conversely, while they are synergistic with RDV, the *C*_max_/EC_50_ ratios for the HCV drugs against SARS-2-CoV are <0.1. (These drugs are more potent against HCV.) The reported synergies of the HCV drugs with RDV do demonstrate the potential utility of combinations targeting the SARS-CoV-2 polymerase and its proteases with DAAs (e.g., MPV plus Paxlovid [18, 22]). Other new oral CoV protease inhibitors (22, 112, 113, 125–127) and polymerase inhibitors (97, 128, 129) should also be considered if they have suitable *C*_max_/EC_50_ ratios. Similarly, new TMPRSS2 inhibitors being explored (130–134) may have more suitable PK properties than camostat.

### Evaluate the half-life, selective index, drug-drug interactions, and side effects of the drug.

Drug toxicity as a function of drug concentration, for example as detected with SynToxProfiler (135), should be considered, as it could limit the effectiveness of a combination found to be synergistic *in vitro* and otherwise suitable for human trials. In addition to known associations with end organ damage, potential positive or negative adjunct effects, such as suppression of cytokine storms and inflammation associated with serious disease, should also be considered. Expedited FDA consideration of two previously approved drugs in combination for an investigational new drug (IND) application requires preclinical evidence of lack of drug-drug interaction, favorable PK/PD, and lack of presumed overlapping toxicities.

### Animal model testing.

Once a list of drug combinations is prioritized, promising cocktails should be tested in a small animal model. The model should approximate human studies in which an agent might be given as postexposure prophylaxis or as early treatment several days after viral inoculation given that efficacy may differ for these two indications for a given regimen. Readouts in animal studies should always include frequent viral load testing in addition to clinical and pathological scores such that early attempts at establishing mechanistic correlates of efficacy can be made.

### Mathematical modeling for regimen optimization.

Mathematical modeling can be used throughout the development of combination antiviral regimens to maximize the likelihood of successful drug, dose, and dosing interval selection, as well as critical features of study design. Mathematical models are predicated on the concept that PK/PD equations are necessary but not sufficient for forecasting trial outcomes. Of equal importance are equations that capture viral and immune dynamics in the absence of therapy (136).

Several principles illustrate the importance of this approach. First, the timing of therapy may predict its effectiveness. In the case of SARS-CoV-2, we determined that intense innate immune responses severely limit the extent of viral replication after the first 5 days of infection (16). Therefore, agents started during the presymptomatic phase of infection, which would parallel postexposure prophylaxis in clinical practice, require higher potency to induce viral clearance than those given later when immune mechanisms assist in clearance of infected cells. Yet, because the majority of viral replication occurs early during infection and is linked to downstream aberrant inflammation, treatment given soon after development of symptoms is critical toward preventing severe outcomes. This model prediction was subsequently validated in treatment trials with MAbs, demonstrating that administration is more effective prior to hospitalization (11–13, 137).

Second, as described above, *in vitro* assessments of potency may overestimate a drug’s antiviral effect in people by 5- to 10-fold (122). Serial viral load measurements in animal models or in human clinical trials, coupled with mathematical modeling are vital to link plasma drug concentrations with viral kinetic outcomes. The goal is to identify the *in vivo* EC_50_, or plasma concentration of drug that eliminates 50% of cellular infection events *in vivo*.

Third, modeling provides necessary context for the potential benefits of combination effects that are additive, multiplicative, or synergistic. By accounting for nonlinear drug levels over time, modeling can capture the proportion of time during which levels of both drugs allow potent inhibition of cellular infection, either by virtue of single drug potency or synergy. This is vital because it can identify scenarios in which addition of a second drug is required to achieve adequate viral suppression and those in which a second agent may be unnecessary and confer unnecessary toxicity.

Finally, modeling can capture possible differential impacts of therapies in various hosts. Whereas most infected people, even those with critical illness, appear to eliminate high grade viral replication within 1 to 2 weeks (138), immunocompromised hosts can shed infectious SARS-CoV-2 at high viral loads for months (60–65), taking on a phenotype more consistent with chronic, persistent viruses like HIV. Here, the virus undergoes considerable mutation and is more likely to become resistant to small molecule DAA or MAb therapy. Immunocompromised hosts are a possible source for VOCs that have dramatically extended the duration and overall lethality of the epidemic (60). Modeling is well poised to capture the added potency required from synergistic agents in this specific clinical context.

Modeling is ideally linked to all steps in the drug development process. PD models are used to identify whether drug pairs have antagonistic, additive, multiplicative, or synergistic antiviral activity at given concentrations and to precisely recapitulate the degree of viral inhibition across all possible ranges of dual drug concentrations (48). PK models recapitulate drug levels over time in relevant animal models or humans. Models can then project the percentage of cell infection events prevented with a given regimen at a given dose. While these calculations allow for an initial estimate of *in vivo* benefits of synergistic or multiplicative effects, the accuracy of forecasts is limited if *in vitro* assays overestimate *in vivo* potency. We therefore fit our models to detailed virologic data from animal studies or early human clinical trials and solve for the *in vivo* EC_50_ (121, 122). We can leverage this information to synthesize PK/PD models with viral dynamics models to arrive at lowest doses and dose frequencies of drug pairs that are likely to have suppressive antiviral efficacy in human trials.

## NEW DRUGS TO CONSIDER FOR COMBINATION TESTING AGAINST CoVs

While approved drugs have the advantage that FDA guidance on the development of drug combinations allows for more rapid development (https://www.fda.gov/media/80100), drugs that have passed human safety trials should also be considered as information about them becomes available. In this respect, it will be important to follow the development of AT-527 (128), other potential oral SARS-CoV-2 polymerase inhibitors (97, 129) including oral forms of RDV (139–141, 200) as well as the oral protease inhibitor GC-376 (102, 125). As new oral/inhaled drugs with the same targets but better efficacy and PK become available, they should be analyzed as substitutes in previously characterized drug synergies. Other drugs for future combination tests could be oral/inhaled variants of effective drugs currently only suitable for injection (142) and drugs of interest in other countries (https://www.ema.europa.eu/en/human-regulatory/overview/public-health-threats/coronavirus-disease-covid-19/treatments-vaccines/covid-19-treatments). Inhaled drugs currently under consideration for SARS-CoV-2 include budesonide (143), ciclesonide (144), interferon alpha (86, 96), interferon beta (145), nafamostat (146), and niclosamide (147), as well as small inhalable biologics, including minibinders (148), nanobodies (149), and peptide fusion inhibitors (150). Many other potential anti-CoV drugs are also being uncovered through *in silico* protein docking, transcriptional profiling, and protein-protein interaction network analyses. An outpatient therapeutic regimen that combines inhaled and oral drugs is plausible. Lastly, future drug combinations could include immune boosters such as STING (stimulator of interferon genes) activators (151, 152) and may be composed of three or more drugs (49–51, 68), if enhanced synergy or PK benefit is projected. As new drug combinations are explored to combat CoVs, a unified set of mathematical equations could allow rapid updates such that new potential regimens are ranked according to their likely potency, as has been done for HIV (50).

## CLINICAL CONSIDERATIONS

Executing high-quality clinical trials during both the early and mature phases of a pandemic presents challenges related to testing of drug combinations, especially for drugs with easily accessible routes of delivery. In the initial phases of an epidemic, tension between conducting research and providing clinical care when resources are constrained means that experimental therapies are often given in uncontrolled studies or as expanded access, although such use “often undermines fair access to experimental agents, [and] compromises the collection of robust data to determine the safety and efficacy of interventions” (153). During the 2014–2017 Ebola epidemic, an advisory panel to the WHO provided a seven-point list to guide conditions of investigational agent use, which urged minimal interference with the conduct of high quality clinical investigations (https://www.who.int/ebola/drc-2018/notes-for-the-record-meuri-ebola.pdf). During the COVID-19 pandemic, widespread clinical use, including through nonprescribed access, and uncontrolled studies has operationally precluded high-quality clinical trials for several repurposed agents, including hydroxycholorquine, ivermectin, and fluvoxamine (154, 155) (NCT04668950, NCT 04885530, and NCT 04510194, but see reference 156 for fluvoxamine). Numerous other small studies used various doses and combinations of proposed synergistic drugs, with each underpowered to meaningfully assess efficacy. It was difficult to meaningfully compare efficacy across disparate study regimens for pooled analysis. Lower regulatory burden improves the speed and cost at which studies on previously approved drugs can be done, but in many cases during 2020, the rapid conduct of such trials impeded progress toward interpretable data.

Presently, at the more mature stage of the pandemic, new hurdles are hampering development of oral/inhaled treatments for SARS-CoV-2. The logical positioning of combination oral drug trials is for early treatment of nonhospitalized patients with either mild symptomatic or asymptomatic infection to maximize clinical effect and prevent hospitalizations. Such potential trials now have an added hurdle because MAbs with EUA are now considered standard of care for people at moderate/high risk for hospitalization, which includes the larger share of unvaccinated adults (https://www.covid19treatmentguidelines.nih.gov/). As such, trials of oral/inhaled drug combinations can no longer ethically be placebo controlled (if conducted where MAbs are available). Because participation in clinical trials requires trust in scientific and medical institutions, persons who remain unvaccinated in places with widespread access to COVID-19 vaccines may be less likely to participate in treatment trials, although careful attention to community engagement has shown incredible success with vulnerable communities (157).

Moreover, these therapies were tested in clinical trials and authorized based on efficacy at reducing hospitalization and death. As SARS-Cov-2 vaccinations increase, an increasing proportion of infections are in vaccinated persons, who remain overwhelmingly protected from severe COVID-19 outcomes. However, ongoing studies of non-high-risk patients (vaccinated and/or without significant comorbidities) need to be evaluated for benefit in symptom reduction and/or viral shedding because hospitalizations are too uncommon an outcome to rapidly conduct studies with adequate statistical power, particularly when the comparator group includes the standard of care. For multiple other viruses, including HIV, HCV, cytomegalovirus (CMV), and HSV-2, establishment of virologic surrogate endpoints has dramatically decreased the number of people and the cost and time associated with licensure trials (158–161). As of this publication, there is no accepted definition for success in reduction of SARS-CoV-2 shedding or transmissible virus, nor are there accepted definitions for change in symptom burden or duration. Both symptoms and viral shedding are also blunted in most infections following vaccination, which additionally limits power to detect a treatment effect. In future pandemics, early trials should be designed to allow establishment of virologic surrogate endpoints. To meet this goal requires that studies include daily virologic sampling and detailed daily symptom surveys. This method will allow trialists to evaluate which viral kinetic feature (peak viral load, duration of shedding or viral area under the curve [158]) or constellation of early symptoms may be most predictive of hospitalization or death. Even if this is achieved, changes in incubation period, viral kinetics, and symptoms with different emerging VOCs may necessitate updating of these surrogate outcomes.

Finally, lack of a path toward EUA for novel drugs, or package relabeling for already approved drugs, based on potentially acceptable surrogate outcomes hampers industry-sponsored drug investigation. Drugs without efficacy as monotherapy in phase 2 studies may not progress to combination trials, despite preclinical data that would suggest potential success and may have predicted lack of success of the single agent. Significant alteration in regulatory procedures during a highly lethal pandemic is another necessary step forward.

## PREPARING FOR OTHER EMERGING VIRUSES

Members of 11 virus families are considered of potential high consequence, and it is important to develop oral (and inhaled, for respiratory viruses), thermally stable, inexpensive, pan-family, drug cocktails to combat all of them (118) (Fig. 1). Common features of these category A to C (category A-C) pathogens (https://www.niaid.nih.gov/research/emerging-infectious-diseases-pathogens) can be exploited in therapeutic cocktail design. All families of concern contain single-stranded RNA and are inhibited by one or more of the following polymerase inhibitors: RDV, MPV, favipiravir, or galidesivir (BCX4430). The members of nine of these families, including coronaviruses, are enveloped and hence deliver their genomes into the cytoplasm to initiate replication by a stereotypical membrane fusion process mediated by a fusion glycoprotein (GP) (37, 38). Many of these (e.g., the hemagglutinins [HAs] of influenza viruses and the GPs of arenaviruses) also bind particles to the host cell surface, whereas other viruses contain a separate attachment/receptor binding protein. Both virus attachment and virus fusion are good targets for small molecule intervention (72, 162–166). As polymerase complexes are clearly excellent therapeutic targets (19, 49, 51), a “starter” pan-family cocktail could include a drug targeting a viral attachment or fusion glycoprotein and a drug targeting the viral polymerase. Flaviviruses and togaviruses also encode proteases that, like the SARS-CoV-2 proteases, process their polyproteins during virus maturation (167, 168). Proteases therefore represent additional targets for a subset of category A-C viral pathogens.

The members of 9 of the 11 category A-C virus families (arena-, bunya-, calci-, filo-, flavi-, orthomyxo-, picorna-, rhabdo-, and togaviruses) enter cells through endosomes (38), and hence, the endosomal pathway is a target for their therapeutic intervention (169, 170). Indeed, many drug screens have uncovered endosome-targeting drugs against these pathogens. Endosomal features that can be targeted are virus particle internalization (e.g., via clathrin or macropinocytosis) as well as endosome trafficking, maturation, and composition, including low pH, cathepsin proteases, Ca^2+^ and other ions, and specific lipids (171–173). Even Nipah virus and Hendra virus, which are category C paramyxoviruses that fuse at the plasma membrane, require a low-pH-activated endosomal cathepsin to process their fusion glycoproteins and form infectious particles (174). Furthermore, endosome-targeted drugs could inhibit infections by CoVs in tissues outside the lung (34–36).

While DAAs are considered ideal based on their generally higher potency and selective indices, we posit that targeting host proteins critically involved in the viral life cycle, including drugs that target endosomes for most category A-C viruses, should be considered in combination therapies, especially if we are preparing for the short-term (plan B) for endosome-entering viruses. From a screen of 78 unique pairs, we identified several demonstrating clear synergistic activity against Ebola virus in cell cultures (47). Based on PK and other considerations, two pairs (bepridil plus sertraline and sertraline plus toremifene) were further evaluated for eventual testing in an oral formulation in mice against a lethal Ebola virus challenge (48). The components of these pairs are approved, endosome-affecting drugs; each had previously been shown to protect mice (50 to 100%) in a lethal challenge model when given intraperitoneally (46, 175). In the same study (48), we showed, through mathematical modeling of PK, PD and Ebola viral dynamics, that compared to their individual constituents, both synergistic drug pairs have superior potential to reduce viral loads in humans (48). It will be interesting to see how these drug pairs function compared to their individual components when given orally to mice infected with Ebola virus. Moreover, all three compounds in these drug pairs have also been shown to bind to a pocket in the Ebola virus GP, thereby affecting its stability (176). Hence, some endosome-targeting drugs may also act directly on viral glycoproteins (69, 176–179), i.e., be both HTAs and DAAs. Other host factors to consider for targeting include host proteases that prime viral glycoproteins for fusion (37, 38, 119, 120), other host proteins involved in virus entry (180), host cell kinases (181–184), and host proteins involved in viral RNA production (185, 186), nuclear export (187, 188), and virus assembly and egress (189, 190). Given common viral infection strategies, the possibility exists for cross-family drug cocktails.

While investigators are tailoring novel drugs to specific viral proteins, we urge that concurrent work proceed to develop cocktails against all category A-C viruses, which might comprise approved (repurposed) drugs or combinations of advanced clinical stage and repurposed drugs. If a new pandemic emerges in the next few years or if resistant VOCs emerge during the current pandemic, individual suboptimal agents with good projected efficacy based on mathematical modeling assessment of synergy and projected drug levels could be tested rapidly, in combinations, in clinical trials. Even a cocktail with incomplete suppression of viral replication could have a clinical benefit: for Ebola virus (191, 192) and SARS-CoV-2 (193), a 1-log-unit-lower viral load has been associated with survival. Reductions in viral loads may also have public health benefits by reducing transmission rates and the opportunity for new variants.

## CONCLUSIONS

SARS-CoV-2 is projected to remain in circulation in the human population, and novel CoVs may spill over from animals to humans (194, 195). An urgent goal is to develop inexpensive oral and/or inhaled regimens to test at the inception of the next outbreak. Drug combinations should be considered in this pursuit to limit drug resistance and enhance potency (Fig. 1). Moreover, the entire antiviral drug arsenal requires significant boosting for global viral pandemic preparedness. It is our opinion that this effort could be significantly augmented by carefully designed drug combination studies, at both the preclinical and clinical stages.
